# The Epital Care Model: A New Person-Centered Model of Technology-Enabled Integrated Care for People With Long Term Conditions

**DOI:** 10.2196/resprot.6506

**Published:** 2017-01-16

**Authors:** Klaus Phanareth, Søren Vingtoft, Anders Skovbo Christensen, Jakob Sylvest Nielsen, Jørgen Svenstrup, Gro Karine Rosvold Berntsen, Stanton Peter Newman, Lars Kayser

**Affiliations:** ^1^ Department of Internal Medicine Q, Bispebjerg Frederiksberg University Hospital Copenhagen Denmark; ^2^ Aalborg University Aalborg Denmark; ^3^ Section of Social Medicine Department of Public Health University of Copenhagen Copenhagen Denmark; ^4^ The Municipality of Lyngby-Taarbaek Lyngby Denmark; ^5^ EmpowerMind Danmark Søborg Denmark; ^6^ Norwegian Center for eHealth Research University Hospital of North Norway Tromsø Norway; ^7^ School of Health Sciences City, University of London London United Kingdom

**Keywords:** integrated care, technology enabled, innovative care, chronic obstructive pulmonary disease (COPD), frail

## Abstract

**Background:**

There is worldwide recognition that the future provision of health care requires a reorganization of provision of care, with increased empowerment and engagement of patients, along with skilled health professionals delivering services that are coordinated across sectors and organizations that provide health care. Technology may be a way to enable the creation of a coherent, cocreative, person-centered method to provide health care for individuals with one or more long-term conditions (LTCs). It remains to be determined how a new care model can be introduced that supports the intentions of the World Health Organization (WHO) to have integrated people-centered care.

**Objective:**

To design, pilot, and test feasibility of a model of health care for people with LTCs based on a cocreative, iterative, and stepwise process in a way that recognizes the need for person-centered care, and embraces the use of digital technology.

**Methods:**

The overall research method was inspired by action research and used an agile, iterative approach. In 2012, a living lab was established in a Danish municipality which allowed for the freedom of redesigning health care processes. As the first step, a wide group of stakeholders was gathered to create a layout for the reorganization of services and development of technology, based on established principles for innovative management of people with chronic conditions. The next three steps were (1) a proof of concept in 2012, (2) a pilot study, and (3) a feasibility study from 2013 to 2015, in which a total of 93 chronic obstructive pulmonary disease (COPD) patients were enrolled. Citizens were provided a tablet-based solution for remote follow-up and communication purposes, and access to a 24/7 response and coordination center that coordinated both virtual and face-to-face support for COPD management. In step five the initial model was extended with elements that support continuity of care. Beginning in the autumn of 2013, 1102 frail elderly individuals were included and offered two additional services: an outgoing acute medical team and a local subacute bed function.

**Results:**

Based on the findings from the iterative process, and evolving technology and workflow solutions, we propose a robust and feasible model that can provide a framework for developing solutions to support an active life with one or more LTCs. The resulting Epital Care Model (ECM) consists of six stages, and serves as a template for how a digitally-enhanced health service can be provided based on patients’ medical needs. The model is designed to be a proactive, preventive, and monitoring health care system that involves individuals in the management of their own health conditions.

**Conclusions:**

The ECM is in accordance with WHO’s framework for integrated people-centered health services, and may serve as a framework for the development of new technologies and provide a template for future reorganization.

## Introduction

For more than half a century it has been recognized that people living with chronic illness need to develop the knowledge and understanding of their conditions. This knowledge provides patients with the means to recognize the appropriate changes in behavior that will improve self-management of their conditions with support from their doctors, allied health professionals, and others in their environment [1,2]. In response to this need, more effective approaches for people with long-term conditions (LTCs) have been developed. One example is the Chronic Care Model (CCM) [3], which was further extended by the World Health Organization (WHO), resulting in the Innovative Care for Chronic Conditions (ICCC) Care Model [4]. During the last decade, many health care organizations have published experiences of reorganization, new business models, new frameworks, and various levels of service transformations to improve care for those with LTCs [5-11].

The main focus of most of these initiatives is to reorganize the way that health care is provided, support people in the management of their own conditions, incorporate caregivers into supporting care, and use a more holistic approach to care [12,13]. This paradigm shift has led to a redesign of services, new tasks for the workforce, and integrated care programs covering different sectors to achieve alignment of programs across sectors, involvement of patients and their relatives, and moving initiatives closer to people’s communities. Although a number of initiatives inspired by the CCM and ICCC have been implemented, the benefits of system reorganization and incorporating the full advantage of digital health remains to be demonstrated on a larger scale.

**Figure 1:**
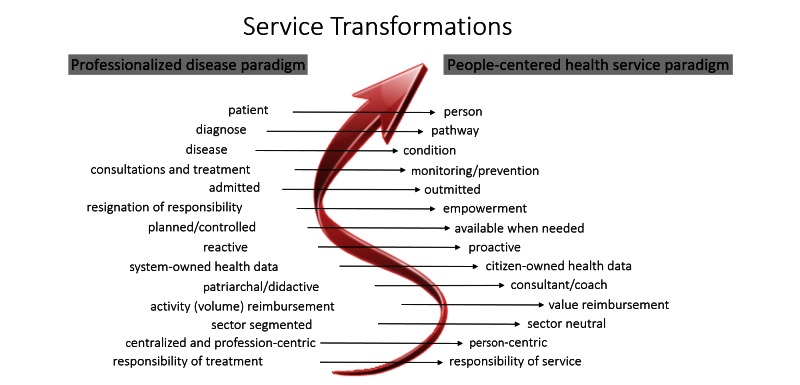
A roadmap for service transformation in healthcare systems. To assist both the individuals and their health care providers, there is an emergent need to develop advanced information and communications technology systems and services for personalized care that may support such transformations.

In response to this need, we present a project that has the overall aim to fill this gap by demonstrating how the entire care process can be redesigned with electronic health (eHealth) tools. This paper outlines the voyage from vision and ideology to a translation of fully functional transformed health care service. Our idea of a person-centric health care system based on the *personalized health paradigm* follows the road map of service transformations shown in Figure 1. The final result aims to be a proactive, preventive, and monitoring health care system that involves individuals managing their own health and conditions.

The overall goals of this project are to (1) provide an understanding of how provision of health care can be designed for people with LTCs by taking advantage of technology, and then (2) based on these findings, propose a new prehospital care model. To achieve the first goal, the first part of this paper, the Methods section, is organized as a step-wise process in which eHealth technology is systematically brought into the design, pilot, and feasibility phases in the development of a novel eHealth driven socio-technical care system. In the second part of this paper, the Results section, we present the Epital Care Model (ECM), which has emerged from the project.

## Methods

### Establishment of a Living Lab

The ECM project was initiated in early 2011, based on knowledge gathered during two previous projects: The Capital Region of Denmark’s development of clinical digital paths for chronic conditions (Kronikerprojekt 5; *K5*) and the *Virtual Hospital* [14,15].

K5 was initiated by the Capital Region of Denmark in 2010, and supported by the Danish Ministry of Health. This initiative resulted in a service model for how three chronic conditions (chronic obstructive pulmonary disease [COPD], ischemic heart disease, and type 2 diabetes mellitus) could be planned digitally. The study demonstrated that it was possible for all health professional stakeholders to interact and coordinate via a digitalized individual plan that contained an updated list with planned health services in a cross-sectorial setup [14]. The model was tested with prototypes and clinical users representing all sectors [14].

In the *Virtual Hospital* study, patients with acute exacerbation of COPD were admitted to a hospital within 24 hours and were randomized to be either *admitted* or *outmitted* [15]. The results suggest that rather than being hospitalized, COPD patients can be treated at home using telehealth technology in combination with an organizational and service redesign, without increasing mortality. The study also demonstrated that individuals need introduction to new ways to manage their condition before they become acutely ill and distressed. Furthermore, if patients are introduced to enhancing technology that increases autonomy and coping, patients are (to a certain extent) reluctant to part with these aides [16].

Inspired by these findings and two other Danish projects [17,18] that demonstrated the benefits of telemedicine support for COPD patients, we assembled a group of stakeholders from the municipality of Lyngby-Taarbæk, in the Capital Region of Denmark. The goal of this initiative was to develop a living learning lab isolated from the existing conventional health care systems, which allows for innovative and disruptive thinking on how to deliver health care [19].

In Denmark, health care provision is organized in three structures: the regions, that run the hospitals and contracts with general practitioners (GPs); the municipalities, that have the responsibilities of rehabilitation and home care; and GPs, who are private doctors working by contract for the regions, taking care of a certain number of people in their community. GPs can be organized in single or group practices [20]. The management of people with chronic conditions is constantly evolving, with strategies being updated every year. The latest update was published in 2016 and is based upon seven recent shared-care projects, including the project reported here [21]. Currently, people with LTCs are handled according to regional clinical pathways, which are designed in accordance with national guidelines [22]. The GP is the coordinator and primarily sees the patients. The hospitals follow the people with more severe conditions and the municipalities are only involved for people with a need of home care, or those who are referred to a standardized rehabilitation program.

The technological infrastructure for national health care is based on a backbone governed by the Danish Health Care Communication Network (MedCom), which is a national infrastructure that enables standardized communication between all official health organizations [23]. MedCom offers a set of standardized communication services, such as a referral from the GP to the hospital or a discharge letter from the hospital to the GP. Additionally, patient data is presented in a national health portal [24], which offers patients and their relatives access to health data and information about clinical conditions with three days of delay. The municipalities have their own care records, which exchange data with the hospital systems and GPs via MedCom communication services. The messages are exchanged in EDIFACT or XML formats. Most health care services are free for everybody and the economy is governed by rates for the hospitals and a fee for service reimbursement system for the GPs. The municipalities also reimburse 34% of the hospitals’ costs related to patient admissions (up to 2000 Euros). A reduction in outpatient visits or patient admissions will therefore result in savings for both hospitals and municipalities. These saving may not materialize for the hospitals. Conversely, a reduction of hospital bed days may increase the costs for the municipalities due to patients spending more days in their own homes, thereby increasing needs for homecare.

### The Theoretical Approach

This project was initiated as a collaborative learning process with noncontractual participation from enterprises and patient associations. Access to a relevant clinical setting was provided through an agreement between the first author (KP) and the mayor of Lyngby-Taarbæk municipality in 2011. The project was designed to be an iterative process, aimed to create collaborative learning, performed by practitioners and with the researchers taking an active role in the project. The process was inspired by action research [25], the agile manifesto [26] with the Scrum model, and the *plan-do-check-act* approach [27], which all build on the same core principles: plan, action, observe, evaluate, reflect, and then iterate. The final stage resulted in a model of how people with LTCs can be managed in a prehospital setting. Originally the project was designed to have four steps, but during this project we decided a fifth step was needed.

### The Process

#### Step 1: Initialization

The initial objectives of the ECM project were

1. To introduce methods, technology, equipment, and organization to support COPD patients to detect potential exacerbation of their disease, in order to establish appropriate early intervention.

2. To set up a management system for acute exacerbations, primarily based on virtual monitoring and remote communication facilitated by telehealth technology, in order to supervise and support self-management.

3. To involve other health care providers in a way that supports explicit patient needs, and is not constrained by official roles or affiliations, to ensure a seamless patient pathway.

4. To design all services to support self-directed, health-literate, and empowered people with COPD.

The team represented: conventional health care services; small, medium, and large enterprises; patient representatives; and researchers. The participants formed an informal network whose goal was to develop a people-centered digitally supported environment that could provide health services, health care, and other resources to support people to take care of their own health. The project was initially limited to COPD, as it is a condition in which an abundant experience with telehealth solutions exist and deteriorations are easy to follow with telemonitoring equipment [28]. The project was organized within the organizational setting of the municipality of Lyngby-Taarbæk.

In 2012, the organization consisted of a communication center that managed the home care of frail patients referred to home care and responded to their alarms. The center did not offer interaction regarding clinical conditions or home monitoring. All medical issues were referred to the patients’ GPs, emergency services, or hospital services.

All participants were invited to weekly meetings and interest groups were formed with specific focuses (eg, empowerment, technology, and research); this process is reported elsewhere [19,29]. A number of enterprises committed themselves to the development of an information and communications technology (ICT) solution to monitor and register data. The resulting platform served as the infrastructure for the proof of concept (POC).

#### Step 2: Proof of Concept

The POC was a platform to test and develop the usability of the digital solution. This testing was carried out together with seven people with COPD from May to July 2012. The digital literacy of participants was also evaluated and usability tests were performed [30].

The telemonitoring devices used in the POC were the same as the ones used in the *Virtual Hospital* study [15]. An algorithm based on the self-monitored data set of the persons/patients was developed to detect deterioration of the condition using a traffic light format. Variation in patterns triggered attention to the nurses in the communication center by a shift in the color code: green, yellow, or red. A red color of current condition then caused a further action, comprised of an examination by a virtual contact to the person with a detailed analysis of the underlying data. If needed, the electronic doctor (eDoctor) was involved in supervision to initiate one of three medical interventions, depending on the severity of the exacerbation.

#### Step 3: Design of Feasibility Study

**Figure 2:**
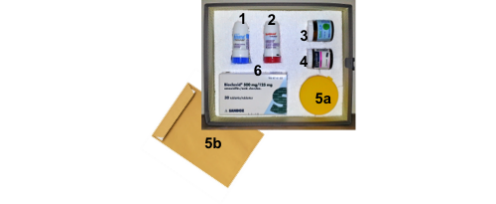
Prototype of medical first aid box containing (1) short acting beta2-agonist for inhalation, (2) corticosteriods for inhalation, (3) anxiolytics (tablets), (4) prednisolone (tablets), (5a) container for sputum sample, (5b) envelope for sending sputum sample, and (6) antibiotics.

The design of the feasibility study was based on inputs from the POC, as well as stakeholders, and was initiated at the end of 2012. The response to the POC involved health care staff and entailed the adjustment of the user interface for those with COPD, and training of nurses who received calls at the existing call center. These nurses and others organized the Response and Coordination Center (RCC) functionality and developed a prototype of a medical first aid box (see Figure 2) with five different medications for treating acute exacerbations. Ordering, delivery, and renewal of medication packages was streamlined as a new service together with the local pharmacies, as was the first step of an electronic prescription.

#### Step 4 (A & B): Kick-Off and Feasibility Study

This project was based on a RCC with access to an on-call pulmonary specialist and equipment designed for home monitoring that was delivered to the participating citizens. The organization and development was based on an iterative process with the aim to support citizens with COPD in managing their own health and in handling exacerbations. The core components were

1. A tablet with software enabling contact with the RCC by video conference, and visualization of the citizen’s condition by algorithms showing indicators of deterioration with an auto-generated written feedback system.

2. Equipment for participants to monitor their own health (eg, spirometer, pulse oximeter).

Box with medicine to be used when exacerbations occurred, to avoid delays in initiation of treatments.

3. 24/7 RCC nurse with access to an eDoctor specialized in respiratory medicine.

4. Optional services by referral (eg, health coaching, dietitian, physiotherapist supporting and motivating behavioral change, and setting of new goals).

The project included services to support the processes, the RCC, and the person with COPD:

1. An eDoctor service for all patients, and to support the RCC nurses.

2. An e-technical service team, for installation, patient education, and support of the equipment.

3. Pharmacies responsible for delivery of medication boxes and advice.

4. An empowerment network consisting of a small sized personal health coaching enterprise, a dietician, and a physiotherapist from the municipality. Each member was available for consultations on a weekly basis.

All services were connected by a software platform that had interfaces designed for both participants and health professional users. In January 2013, staff were trained and the services were organized. Recruitment through rehabilitation centers, patient associations, and leaflets resulted in the first participants being enrolled in April 2013. The feasibility study continued until December 2015.

A total of 93 persons with COPD were recruited, educated, and followed (32/93, 34% male; 61/93, 66% female; mean age 73.9 and mean forced expiratory volume = 1.1 liters/second). The patients were included after a consultation by the eDoctor was performed at the patient’s home, including a clinical examination and a stratification of their COPD in accordance with the Global Initiative for Chronic Obstructive Lung Disease guidelines and a subsequent evaluation by the e-technical service team. The participants were only included if they were deemed eligible, based on an evaluation of their cognitive function (Mini-Mental State Examination score above 22) and were able to cooperate and communicate with the technology.

The services provided support for active and independent living. When needed, or due to exacerbations of symptoms, more interaction with the RCC occurred with both nurses and eDoctors, who were available by virtual connection. Participants could monitor their health when they chose to, and were able to discuss their condition with nurses who were supported by the eDoctors. This approach led to a degree of active and independent living, which for some of the participants resulted in a new experience of freedom, so much so that several participants felt able to leave their homes and even travel.

As a result of the feasibility study, the following changes were made: (1) the software for the user interface was programmed as open source to enable dissemination of the code without license restrictions; (2) a second generation of the software was developed as a cocreative process between clinicians and vendors; and (3) an eDoctor clinic was established to have a legal point of entry for the medical doctors, prescriptions, and diagnostic work in this cross-sectorial setting.

#### Step 5: Extension of Prehospital Care Services

By the end of 2013, it was clear that the eHealth organization that had been developed had the capacity to include more extensive services than just COPD follow-up. In the last (and unanticipated) phase, we developed a new eHealth-supported service that provided in-home clinical evaluation and treatment for the following frequent conditions among frail elderly patients: urinary infection, pneumonia, COPD in exacerbation, dehydration, constipation and diarrhea, and confusion and dizziness.

The service structure was in place in September 2013, and was enhanced to include four in-house Subacute Surveillance Beds within the municipal care center, and in June 2014 also included a Mobile Acute Team (MAT). In addition, a business case was developed to convince local politicians that the project had the potential to be cost-effective, as it would reduce the number of hospitalizations by more than 40%. The business case builds on unpublished data from another project led by the first author (KP) at the Frederiksberg Hospital.

### The Mobile Acute Team

This new service sent MAT nurses to the frail participants’ homes when acute problems were detected. The team included five specially trained nurses certified to manage the conditions mentioned above. The eDoctors from the project developed clinical guidelines for the team based on the Capital Region’s guidelines for the management of hospitalized patients, which was adapted to settings outside the hospital.

Individuals in need of a clinical evaluation could be referred to the MAT team by GPs or nurses in the municipality service, or those located at nursing homes. Those patients referred were offered an evaluation that included a medical history, clinical examination, registration of early warning score, paraclinical tests with routine blood samples and blood gas analyses, and collection of sputum and urine for investigation. The samples were collected on-site by the MAT nurse and sent to a small municipality lab for analysis. All results were immediately discussed with the eDoctor.

The MAT can administer intravenous fluids for rehydration, initialize peroral medical treatment in collaboration with the eDoctor service, and administer nebulizers for inhalation therapy for COPD. The MAT service resulted in four different clinical paths: (1) finalizing the evaluation and treatment, and referral to the participant’s GP for follow up; (2) a follow up plan for a period, in which the participant is regularly evaluated by the nurses and an eDoctor; (3) referral to the Subacute Surveillance Place (SAP) unit, for participants to have immediate access to a health professional, be evaluated regularly, and see a doctor within 24 hours; or (4) referral to a hospital for more thorough assessment and treatment.

### The Subacute Surveillance Place

The SAP had the main purposes of being an alternative to admission to hospital, and having the potential of increasing quality of life for participants with acute deteriorations. The goal was to train and increase the competence of the municipality nurses to enable them to take care of uncomplicated medical conditions while being supervised by the eDoctors, with the intention of preventing unnecessary costly hospital admissions. Four in-house beds were established and five nurses were certified to handle the treatment, observation, and care of the patients (based on guidelines).

Upon arrival, patients went through a standardized medical examination, including history and clinical examination by the nurse, and supplemented with paraclinical tests. The results were confirmed with the eDoctor and relevant treatment regimens were initiated. Within 24 hours the patients were physically seen by a medical doctor for further planning.

Between September 2013 and December 2015, 168 participants were admitted to the SAP, and the MAT team performed 2007 home visits for 1102 different individuals.

### Proposal of a Whole-System Approach for Management of People With One or More Chronic Conditions

The evolutionary process described above was intended to be disruptive, and occurred outside the established system. This experience has led us to propose a new model for organizing and providing health care for people with one or more LTCs. The proposed model is in alignment with Wagner’s CCM, WHO’s ICCC, and the newer WHO framework for integrated people-centered health services [11]. The key characteristics are to provide accessibility with a model that is not overly complex, and is intended to be less costly than current models.

## Results

**Figure 3:**
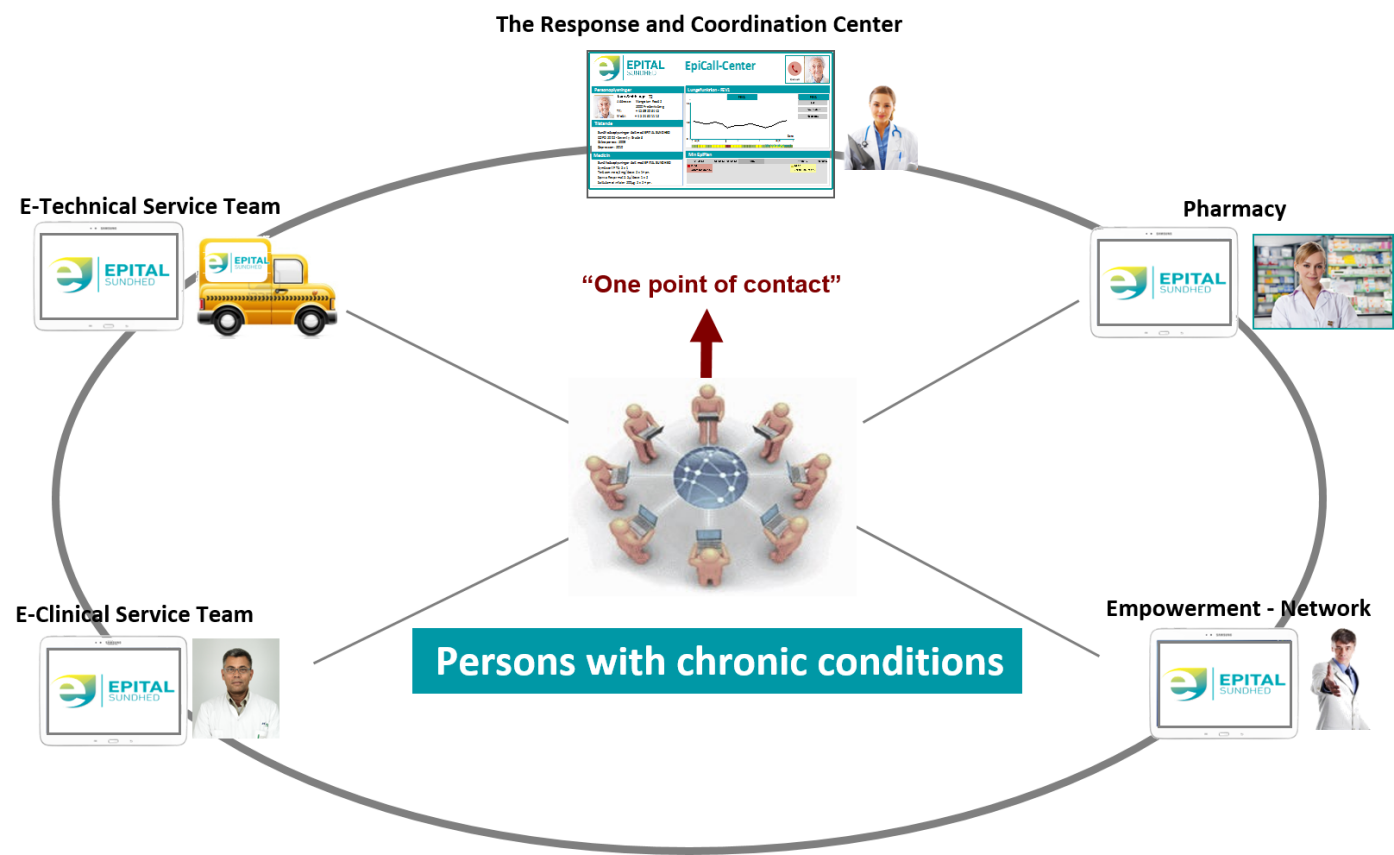
Epital Care Model services in the proof of concept and feasibility study.

Figure 3 outlines the principles of the ECM, and can be conceptualized as a step-wise increase in the care resources that are made available in response to increased care needs. All care is person-centered, which in the ECM means that the patient is in control of care goals, care plans, and changes in care plans.

Each stage of the funnel builds on the former stages. The patient will move back and forth in the funnel depending on their current health condition. The patient voluntarily monitors his/her health condition, and transmits their health status to the RCC by ICT-communication on the mobile tablet. The tablet supports relevant sensors, self-reports, and video communication with the RCC. The RCC responds to changes in health status, according to the agreements made with the patient, as they are recorded in their individual care plan. The RCC responds and triages unanticipated or emergency requests together with the patient, with a primary focus of uncovering the citizen’s and informal caregiver’s needs.

### Stage 0: Citizens With Undiagnosed/Unknown Long-Term Conditions

This segment of the population is invisible and therefore not connected to the ECM service network. The focus here is on early detection of potential diseases. The GPs and patient organizations/networks are likely to play an essential role at this stage.

### Stage 1: Active and Independent Living

In this stage citizens live their life connected to the ECM services in accordance with their personal needs, values, and preferences. This stage is possible even in the face of serious health issues. Support for the active informed patient, in terms of self-management support, is fundamental both here and at all other stages of the funnel. The local pharmacies offer regular evaluation of prescriptions for enrolled individuals. Enrolled citizens have an acute medicine box with prescribed medication for acute exacerbations, which allows the eDoctor to initiate urgent treatment at home. The participants are offered free use of the empowerment network services.

### Stage 2: Virtual Assisted Living

The participant makes use of virtual support through *one-point-of-contact* with immediate response and 24/7 availability, also including indirect access to the eDoctor *.* The RCC provides e-consultations with relevant health professionals. Proactive treatment may be started using the acute medicine box.

### Stage 3: Virtual Assisted Living With Assistance From Home Care Mobile Health Professionals

**Figure 4:**
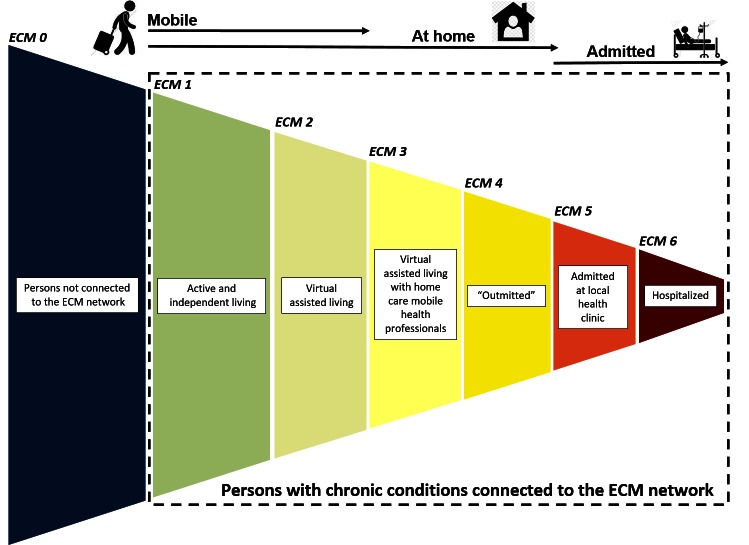
The Epital Care Model illustrated as a funnel.

Virtual assistance is added with mobile support in the participant’s home, such as physical visits from the MAT (see Figure 4). The empowerment network can still be used in this stage, and all stages listed above.

### Stage 4: Outmitted at Home

The goal is to enable the participant to stay in their home as long as possible without compromising safety. Treatment, monitoring, and follow-up by both virtual and mobile teams can be intensified, corresponding to an inpatient setting. The eDoctors play an essential role in this setting, being proactively available virtually and responsible for the treatment in the citizen’s home, while having the MAT nurses as the *eyes and hands* at the point of care in the home. The empowerment network is not activated in stages 4 and 5, in which services are switching towards traditional health care deliveries.

### Stage 5: Admitted at Local Health Clinic

If required, the participant is moved to a local health clinic (SAP) equipped with basic hospital facilities, including 24/7 physical availability of certified ECM health professionals.

### Stage 6: Hospital

**Figure 5:**
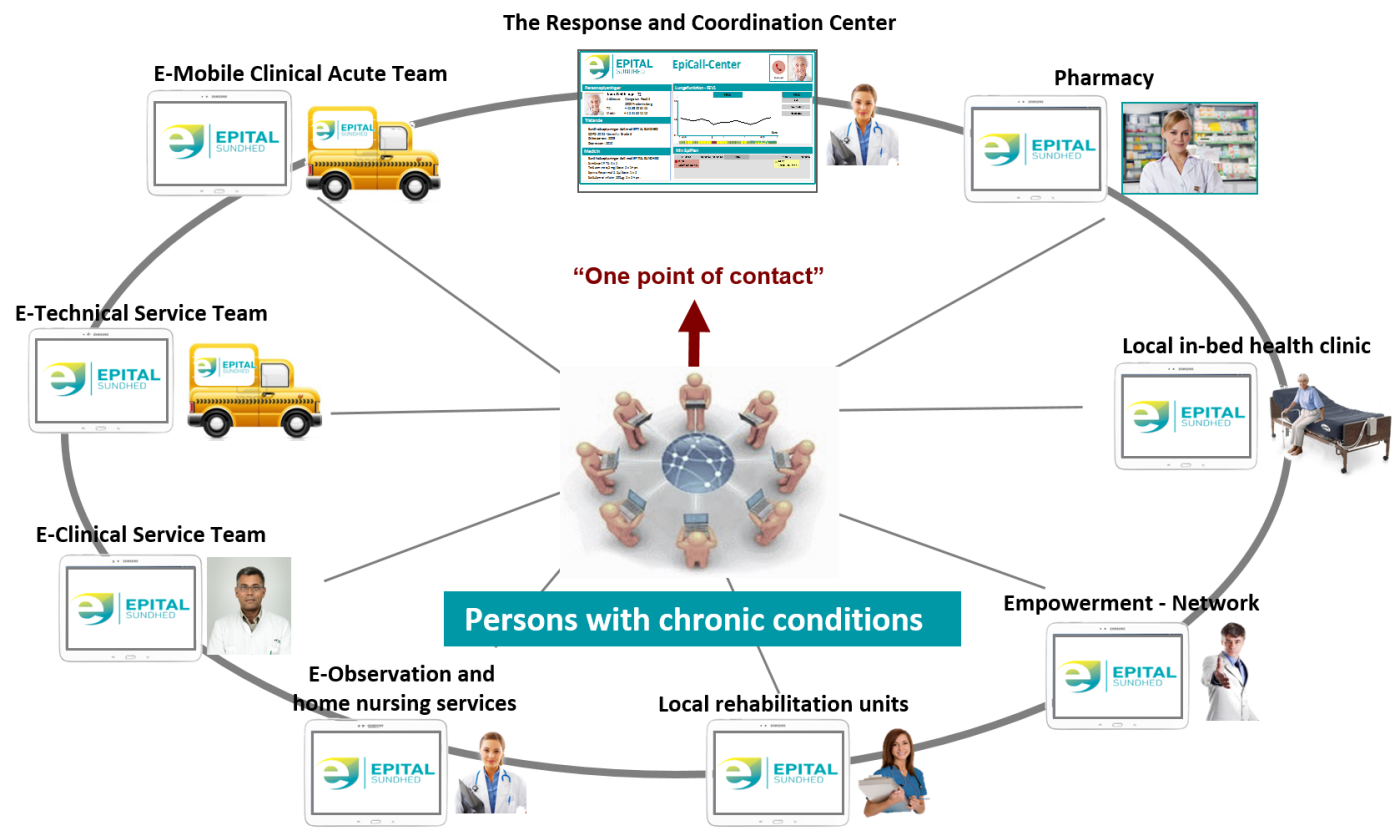
Full scale Epital Care Model services: the health services that are embedded in the Epital Care Model covering ECM 1-5.

If the former services and efforts are insufficient, the citizen is admitted to hospital with specialized health care services. An implementation of the model is the service that is currently integrated into the ECM in Lyngby-Taarbæk, as shown in Figure 5. The concept involves the following organizational elements of health care, all of which are supported by advanced ICT technology:

1. A 24/7 RCC responds to all requests for service and coordinates the delivery of services. The RCC also supports the participant’s plan and is responsible for data coordination. Nurses and social care specialists staff the RCC.

2. Empowerment network services are delivered either as e-services at home through digital tools (ie, training, health coaching, and dietary support), or as personal or group sessions via electronic video communication channels.

3. An e-technical service team is able to support participants’ needs at home with regard to installing technology and training with the technology. The service team also brings medicine cases and collects laboratory tests that are performed at home.

4. The E-Clinical Service Team, including an eDoctor, is responsible for the execution of all clinical services in the total system. Preferably the clinical team works via the electronic channels, but uses the media as necessary to guarantee the best possible support of participant decisions, including home visits. Ultimately, specialist guidance can be available for the citizen, if needed.

5. The pharmacy provides support by disseminating information to participants and training patients in the use of medicine, and produces and delivers the medicine required.

6. An observation service, coordinated by the RCC, which is capable of online observation of measurements from home care instruments connected to the citizens’ home technology. This service is designed to seamlessly take control upon the participant’s request.

7. An outgoing team of specialized nurses provide home nursing. These nurses are skilled in preventive treatment, if health is deteriorating rapidly and threatening the possibility of hospitalization.

8. Local rehabilitation units ensure rehabilitation when wanted and needed.

9. A local in-bed clinic service, for citizens who cannot continue at home.

### Epital Care Model-Compliant Technology Setup

Appropriate technology is a prerequisite for a health care system to become ECM-compliant. The technology must be able to support the core ECM services, the ECM actors, and their collaboration and coordination. As a consequence, various tools, devices, and ICT functionalities must be available for the ECM actors. At all times, it is mandatory to keep an alignment between services, actors, and functionality of the technologies. The core processes of the ECM are briefly described in Table 1.

Actors are persons or digital agents who are characterized by their role, organizational and employment relations, authorization, certification, health literacy, and capabilities. The central actors of an ECM network are described in Table 2.

To execute the ECM services in an easy and appropriate way, the actors must have access to a set of ICT devices and functionalities that are shown in Table 3.

**Table 1:** Core Epital Care Model (ECM) services.

Process type	Short description
Inclusion and stratification	All participants are offered: general health information; clinical examination for diagnosis, stratification, and medical information; and delivery and education in using ECM equipment, including acute medicine box.
Self-monitoring	The self-monitoring process includes the measurement and acquisition of relevant information in context. Examples include recurrent self-monitoring of pulmonary function, temperature, and pulse. These measurements need to be integrated into daily living. The self-monitored data is the fundamental resource that enables all others activities in the ECM network to be stratified.
Supervision	The Response and Coordination Center (RCC) monitors and supervises the connected population; this includes surveillance functions that can detect persons at risk for developing subacute complications and acute deteriorations, in order to initiate preventive and proactive interventions.
Contact	All participants can contact the RCC at any time, or be contacted if the RCC registers any deterioration. The contact can be text-based (asynchronous communication), via telephone, or via videoconference (synchronous communication). Depending on the RCC’s assessment of the situation, a contact may be followed by an advisory consultation, a visit for more assistance, or initiation of medical treatment.
Clinical examination	The clinical examination can be either physical or virtual if video-based communication is possible. The clinical examination will vary in structure but always includes a clinical conclusion based on the content and findings at hand, and will be documented in the medical record.
Treatment	Medical treatment is always preceded by monitoring and a clinical examination. A medical treatment is typically a process involving a prescription of medication and a timeline with follow up consultations, either physically (ECM >2) or virtually (ECM=2).
Pharmacy services	The pharmacy services include ordering, packing, and delivering prescribed medicine, as well as maintenance of the content of the acute medicine box.
Technical service	Technical services include delivery, maintenance, user education, and problem solving activities regarding the ECM-connected person’s equipment.
Paraclinical investigation	Paraclinical services include diagnostic procedures of different kinds (eg, blood sampling and analysis, urine tests, and electrocardiogram).
Health coaching services	Health coach services are consultations (or a series of consultations) for empowering a person.

**Table 2:** Epital Care Model (ECM) actors.

Actor type	Short description
Person	An ECM-connected person is an individual with one or more chronic conditions who is a member of an ECM network. The person gradually moves to be a patient when the context shifts from ECM1 (active and independent living) towards ECM6 (hospitalized living).
Response and Coordination Center nurse	The Response and Coordination Center (RCC) nurse is the coordinator of the ECM network. The RCC nurse conducts population monitoring, initiates proactive contacts to ECM connected persons at risk, and coordinates the services in treatment for acute deteriorations. The RCC nurse is always backed up by a responsible eDoctor regarding prescriptions and decision making.
Mobile Acute Team nurse	Mobile Acute Team nurses are trained and certified in handling frail people, in collaboration with the eDoctors; this includes clinical assessment and investigations.
Subacute Surveillance Place nurse	Subacute Surveillance Place nurses are municipality nurses who are qualified to take care of the patients in the subunit.
eDoctor	The eDoctor is a medical doctor combining a medical specialty (typically general practitioner or internal medicine) with a specialization in eHealth. The eDoctor is capable of taking treatment responsibility in the ECM network, which includes virtual treatments according to the context filters in the ECM funnel.

**Table 3:** Information and communications technology functionality and devices.

ICT/device type	Short description
eHealth-box	The eHealth box is delivered to all Epital Care Model (ECM)-connected persons and contains: (1) an Android tablet with the ECM Health Navigator (Appinux) that contains a condition app (which includes an algorithm for triaging a data set regarding lung function measures) and the ability to video conference with *one point of contact* to the Response and Coordination Center (RCC); (2) a spirometer; (3) a pulse oximeter, included in the facility to measure pulse activity; (4) a thermometer; and (5) a medical first aid box.
Epital Care Model medical record (EpiProcess)	EpiProcess is a process-oriented shared care system that enables the eDoctor and the RCC nurses to input into the same system and coordinate their activities, particularly with respect to exacerbation treatments and follow-ups. EpiProcess is integrated with the Health Navigator, enabling the RCC and eDoctor to evaluate or react on the ECM population’s self-monitored data.
Municipality Care Record (Avaleo)	The municipality of Lyngby-Taarbæk has a care record system (Avaleo) which is used for those who receive municipality health care services. EpiProcess can communicate with the Avaleo system via MedCom messages.
Messaging (MedCom) and the Health Communication Network	All activities that involve medical treatment are electronically communicated to the general practitioner through standardized MedCom messages generated by EpiProcess.
National Medication Service (FMK)	FMK is a national medication service for coordinating updated information about all medicine related to persons [31]. FMK was not available at the time of the feasibility study, and prescribed medicine had to be documented in both the EpiProcess and the eDoctor systems.

## Discussion

For more than half a century it has been realized that people with chronic conditions need to be involved in managing their own conditions through education and activities, which increase health literacy and agency, and facilitate changes in health behaviors [1,2,32]. In 1996, Wagner [3] focused on this issue and since then it has been repeatedly emphasized [4,12,33]. In 2016 the WHO concluded that we are still in need of models and solutions to support large scale implementation of integrated people-centered health services [11].

In this paper, we propose a new way to organize health services for people with chronic conditions. The model is based on a five-year period of action research involving organizational learning and a collaboration between a wide range of stakeholders. The ECM has addressed three great challenges: how to create a personal engagement, how to benefit from using technology, and how health care provision can be reorganized. The ECM integrates our learning from two projects with different approaches into one new model that supports people in all stages of their chronic conditions, and offers them an opportunity for active living. One approach is the involvement of people with COPD in monitoring and handling their condition, which is assisted by an RCC. The other approach is to manage individuals suffering from acute deteriorations with a significant risk for acute hospital admission. The most frequent causes of the acute deterioration include dehydration, infection, and other acute medical problems. Our learning has resulted in a fusion of the different approaches into one model, which may serve as a template or governance model for provision of health care to people with one or more LTCs, including the challenges of those struggling with multimorbidity and polypharmacy.

The ECM meets the requirements set out by Wagner in his proposed model for chronic care, as it both moves services closer to the individual in need, engages the participants, and includes technology to support the use of guidelines and decision making. The certification of nurses, and the way in which they work by delegation, instructions, and in dialogue with the eDoctors, also supports a changed role with new tasks. In this project, we have succeeded with a cocreative process and created organizational learning by including practitioners, academic researchers, the municipality, and other organizations.

The activities in ECM1-ECM3 have proven to be feasible and have been adopted by the involved stakeholders, and recognize that informed individuals should be encouraged and supported to make personal decisions related to their health. These decisions should be guided by their own values, and influenced (but not directed) by the health professionals’ views, as recommended by Zoffmann et al [34,35].

The ECM demonstrates how technology may be used to get the best of the digital and real world to provide continuous care, thereby increasing agency and support for people to have an active and independent life, despite the burden of their chronic condition. To achieve the full benefits in ECM1 and ECM2, each participant must have a unit (such as a smartphone or tablet) with appropriate applications installed to support the management of their conditions. In ECM3-ECM5, the technology primarily supports the health professionals, but may still be used by the participants to communicate with the RCC and the connected caregivers.

It should be noted that the participants in the COPD project reported here were all able to interact with the RCC themselves and did not need support from relatives. A large-scale inclusion of people with LTCs may require that formal or informal caregivers assist them to achieve the full benefits of a given intervention, especially if the participants are cognitively impaired. Other disabilities can often be compensated by built-in accessibility tools in the devices. The training by the e-technical service team may also improve the performance of the participants by addressing particular needs in relation to their digital health literacy [36].

The model developed here may serve both as a template for development of new technologies for people with LTCs, and for future reorganization of health care. The model is generic and is not limited to a particular health organization or service provider, but does require the individual to be actively engaged with services across multiple sectors. The ICT tools of the ECM were developed by three vendors, but the ECM service model is generic and can serve as a framework for other vendors and health care providers. The key is to implement proactive, person-centered solutions based on the principles of the CCM and ICCC, which are enabled by technology.

The innovation process was inspired by Clayton Christensen [19] and it was recognized from the start that this project would be a disruptive influence process working from outside the established system. During the project, the project group experienced restrictions due to legislation and the existing structures for provision of health care. Various strategies emerged, including the proactive and disruptive establishment of an eDoctor clinic, and the more reactive establishment of formal and informal contacts to institutions in the conventional health care system.

The initial intention to create a disruptive model for health care provision resulted in a model influenced by a normalization process [37]. The resulting ECM is not (as initially intended) a model for a parallel health care system, but serves to inform existing health care organizations about how to assist people with LTCs in managing their health in a proactive, people-centered, technology-enabled, and team-based manner. Although it may be difficult to make disruptive changes in a publicly-financed health care system, it is through a disruptive approach that fundamental change occurs in health care, which in turn has the capacity to lead to improved care and reduced costs.

The ECM has emerged from an empirical iterative research process involving public and private partners with wide contacts in the established health care system. The next step is to design a *migration* process of the ECM into other care contexts. This process has already started, and the project might have influenced the Danish national strategy, as the Danish government now focuses on decision support for elderly along with increased involvement of patients [21]. The core principles of ECM are also being used as inspiration for a Norwegian project involving three regional sites [38].

The ECM operationalizes two WHO policy papers [4,11]. It remains to be demonstrated to what extent the ECM will create beneficial outcomes and be economically beneficial. Results from the feasibility study are currently under evaluation. The ECM remains to be proven in a large-scale study that is designed to evaluate the overall benefits to people with LTCs. To facilitate the understanding of the proposed model, please refer to the screencast in Multimedia Appendix 1.

ECM has characteristics that enable the empowerment and engagement of people, reorganization of the provision of care, transformation of roles, and development of more skilled professionals by taking advantage of technology. This approach is in accordance with the intentions of the WHO’s framework for integrated people-centered health services [11]. ECM may therefore be a model to assist vendors, health care organizations, and researchers with populating the WHO framework.

We welcome researchers and/or health care provider organizations to participate in the further development of both the ECM building blocks as well as the ECM. The core of the ECM is that each individual makes their own informed decisions, and are advised by the health care team and the services provided by the health care navigator. Hopefully the ECM, with its framework and an increasing number of health care providers and vendors, will move the health care sector from a specialist-centered, fragmented, patriarchal system to a person-centered, supporting, educating, and cocreative environment. This shift will support people with LTCs in living an independent and active life, and allow them to feel safe with the freedom of mobility. Our goal is not simply related to integrated care, but also relates to personalized care in the context of the individual’s activities and preferences.

## Appendix

Multimedia Appendix 1 Screencast.

## Abbreviations

CCM: Chronic Care Model
COPD: chronic obstructive pulmonary disease
ECM: Epital Care Model
eDoctor: electronic doctor
eHealth: electronic health
GP: general practitioner
ICCC: Innovative Care for Chronic Conditions
ICT: information and communications technology
K5: Kronikerprojekt 5
LTC: long-term condition
MAT: Mobile Acute Team
POC: proof of concept
RCC: Response and Coordination Center
SAP: Subacute Surveillance Place
WHO: World Health Organization

## References

[1] Simonds SK (1963). Health education and medical care: focus on the patient. Health Educ Behav.

[2] Task Force on Patient Education for the President's Committee on Health Education (1974). The concept of planned, hospital-based patient education programs. Health Educ Behav.

[3] Wagner E, Austin B, Von Korff M (1996). Organizing care for patients with chronic illness. Milbank Q.

[4] Pruitt S, Annandale S (2002). Innovative care for chronic conditions: building blocks for action.

[5] Main T, Slywotsky A https://www.hqsc.govt.nz/assets/Consumer-Engagement/Partners-in-Care-Resource-page/PIC-volume-to-value-Oct-2013.pdf.

[6] Thomson S, Osborn R, Squires D, Reed S (2011). International Profiles of Health Care Systems.

[7] Pan American Health Organization (2013). Innovative Care for Chronic Conditions: Organizing and Delivering High Quality Care for Chronic Noncommunicable Diseases in the Americas.

[8] Steventon A, Bardsley M, Billings J, Dixon J, Doll H, Hirani S, Cartwright M, Rixon L, Knapp M, Henderson C, Rogers A, Fitzpatrick R, Hendy J, Newman S, Whole System Demonstrator Evaluation Team (2012). Effect of telehealth on use of secondary care and mortality: findings from the Whole System Demonstrator cluster randomised trial. BMJ.

[9] European Commission (2012). eHealth Task Force Report - Redesigning health in Europe for 2020.

[10] World Health Organization WHO global strategy on integrated people-centred health services 2016-2026: executive summary.

[11] World Health Organization (2016). Framework on integrated, people-centred health services: Report by the Secretariat.

[12] Singh D, Ham C (2005). Improving care for people with long-term conditions: a review of UK and international frameworks.

[13] Ham C, Dixon A, Brooke B (2012). Transforming the delivery of health and social care: the case for fundamental change.

[14] Koncern IT RH (2011). Foranalyserapport: Kronikerprojekt 5 - Demonstrationsprojekt til it-underst&oslash;ttelse af forl&oslash;bsprogrammer.

[15] Jakobsen A, Laursen L, Rydahl-Hansen S, Østergaard B, Gerds T, Emme C, Schou L, Phanareth K (2015). Home-based telehealth hospitalization for exacerbation of chronic obstructive pulmonary disease: findings from “the virtual hospital” trial. Telemed J E Health.

[16] Emme C, Rydahl-Hansen S, Østergaard B, Schou L, Svarre Jakobsen A, Phanareth K (2013). How virtual admission affects coping - telemedicine for patients with chronic obstructive pulmonary disease. J Clin Nurs.

[17] Dinesen B, Hejlesen O, Andersen S, Toft E (2011). Telerehabilitation for COPD patients across sectors: using technology to promote cooperation among healthcare professionals.

[18] Sorknaes A, Bech M, Madsen H, Titlestad I, Hounsgaard L, Hansen-Nord M, Jest P, Olesen F, Lauridsen J, Østergaard B (2013). The effect of real-time teleconsultations between hospital-based nurses and patients with severe COPD discharged after an exacerbation. J Telemed Telecare.

[19] Hesseldal L, Kayser L (2016). Healthcare innovation - The Epital: a living lab in the intersection between the informal and formal structures. Qual Sociol Rev.

[20] Kierkegaard P (2013). eHealth in Denmark: a case study. J Med Syst.

[21] Sundheds- og Ældreministeriet, Sundhedsdatastyrelsen, KL, Danske Regioner http://www.sum.dk/Aktuelt/Nyheder/Digitalisering/2016/maj/~/media/Filer%20-%20dokumenter/Digital-underst%C3%B8ttelse_komplekse_tv%C3%A6rg%C3%A5ende_patientforl%C3%B8b_sammenfattende_rapport%20DOK90723.ashx.

[22] Sundhedsstyrelsen https://www.sst.dk/da/sygdom-og-behandling/kronisk-sygdom/~/media/367F3C29161A4252A631753602638599.ashx.

[23] (2015). MedCom.

[24] Sundhed.

[25] Waterman H, Tillen D, Dickson R, de KK (2001). Action research: a systematic review and guidance for assessment. Health Technol Assess Winch Engl.

[26] Beck K Principles behind the Agile Manifesto.

[27] Bulsuk K (2009). Taking the First Step with the PDCA (Plan-Do-Check-Act) Cycle.

[28] McLean S, Nurmatov U, Liu JL, Pagliari C, Car J, Sheikh A (2012). Telehealthcare for chronic obstructive pulmonary disease: Cochrane Review and meta-analysis. Br J Gen Pract.

[29] Hesseldal L, Kayser L (2016). Healthcare innovation - The Epital: an ethnographic study of a unique way of organizing healthcare innovation. Qual Sociol Rev.

[30] Vang A, Knudsen A http://dias.kb.dk/downloads/dias:128?locale=da.

[31] (2017). FMK-online.

[32] Askham J, Coulter A, Parsons S, Permanand G (2008). Where are the patients in decision-making about their own care?. Policy Brief.

[33] Cruickshank J (2010). Healthcare without walls. A framework for delivering telehealth at scale.

[34] Zoffmann V, Kirkevold M (2007). Relationships and their potential for change developed in difficult type 1 diabetes. Qual Health Res.

[35] Zoffmann V, Kirkevold M (2012). Realizing empowerment in difficult diabetes care: a guided self-determination intervention. Qual Health Res.

[36] Kayser L, Kushniruk A, Osborne R, Norgaard O, Turner P (2015). Enhancing the effectiveness of consumer-focused health information technology systems through eHealth literacy: a framework for understanding users' needs. JMIR Hum Factors.

[37] May CR, Mair F, Finch T, MacFarlane A, Dowrick C, Treweek S, Rapley T, Ballini L, Ong BN, Rogers A, Murray E, Elwyn G, Légaré F, Gunn J, Montori VM (2009). Development of a theory of implementation and integration: Normalization Process Theory. Implement Sci.

[38] Nasjonalt senter for e-helseforskning 3P - Pasienter og profesjonelle i partnerskap.

